# Extramedullary Involvement in T/Myeloid Mixed Phenotype Acute Leukemia With BCR::ABL1 Fusion in a Hispanic Female Patient: A Case Report

**DOI:** 10.7759/cureus.85214

**Published:** 2025-06-01

**Authors:** Camille L Santiago-Negron, José M Cordero Hernández, William D Marrero-León, María D Rivera Rolón

**Affiliations:** 1 Pathology and Laboratory Medicine, University of Puerto Rico, Medical Sciences Campus, San Juan, PRI; 2 Hematology-Oncology, San Juan Municipal Hospital, VA Caribbean Healthcare System, San Juan, PRI; 3 Hematopathology, University of Puerto Rico, Medical Sciences Campus, San Juan, PRI

**Keywords:** acute myeloid leukaemia, cervical lymphadenopathy, hematology-oncology, mixed-phenotype acute leukemia, mixed-phenotype acute leukemia with bcr-abl1 translocation, t-cell leukemia

## Abstract

Mixed phenotype acute leukemia (MPAL) is a rare subtype of acute leukemia characterized by the expression of markers from more than one lineage. The T/myeloid subtype, especially with extramedullary involvement and *BCR::ABL1* fusion, is exceptionally rare and diagnostically challenging. We report a case of a 61-year-old Hispanic female patient presenting with generalized lymphadenopathy. Excisional biopsy of a left occipital lymph node showed diffuse effacement by a monomorphic population of blasts. Immunohistochemistry revealed co-expression of myeloid (partial MPO, lysozyme) and T-lineage markers (CD3, CD4, CD5, CD7). Bone marrow biopsy confirmed MPAL with extensive infiltration by CD3+, CD5+, and CD2+ cells; approximately 20-30% co-expressed CD34, CD117, and TdT. Flow cytometry supported a diagnosis of T/Myeloid MPAL. Fluorescence in situ hybridization (FISH) analysis identified *BCR::ABL1* rearrangement. This case highlights the importance of integrating morphology, immunophenotyping, and molecular testing to diagnose MPAL and underscores the need for clinical awareness of its varied presentations, including extramedullary disease.

## Introduction

Mixed phenotype acute leukemia (MPAL) is a rare and biologically heterogeneous group of leukemias characterized by blasts co-expressing markers of different lineages, including myeloid, B-lymphoid, and T-lymphoid antigens. It accounts for approximately 2%-5% of all acute leukemias and poses significant diagnostic and therapeutic challenges [1,2]. The World Health Organization (WHO) classifies MPAL based on immunophenotypic and molecular features, including subtypes such as B/Myeloid and T/Myeloid [3].

MPAL with a T/Myeloid phenotype is particularly uncommon, and extramedullary involvement, especially with nodal disease, is even rarer. MPAL with *BCR::ABL1* fusion is associated with a poor prognosis and requires targeted therapy with tyrosine kinase inhibitors (TKIs) alongside intensive chemotherapy [4,5]. We report a case of MPAL (T/Myeloid) with *BCR::ABL1* fusion with generalized lymphadenopathy as the primary presentation, emphasizing the histopathological, immunophenotypic, and molecular features crucial for diagnosis.

## Case presentation

Clinical presentation

A 61-year-old female patient presented with progressive, painless generalized lymphadenopathy. Physical examination revealed palpable lymph nodes in the cervical, axillary, and inguinal regions. She denied constitutional symptoms such as fever, night sweats, or weight loss. Laboratory evaluation showed significant leukocytosis (white blood cell count (WBC) 75.2K/µL) with circulating blasts (45%) and lymphocytosis (42%). Normocytic-normochromic Anemia (hemoglobin (Hb) 10.8 g/dL) and (platelets 211K/µL) were also noted.

Histopathology

**Figure 1:**
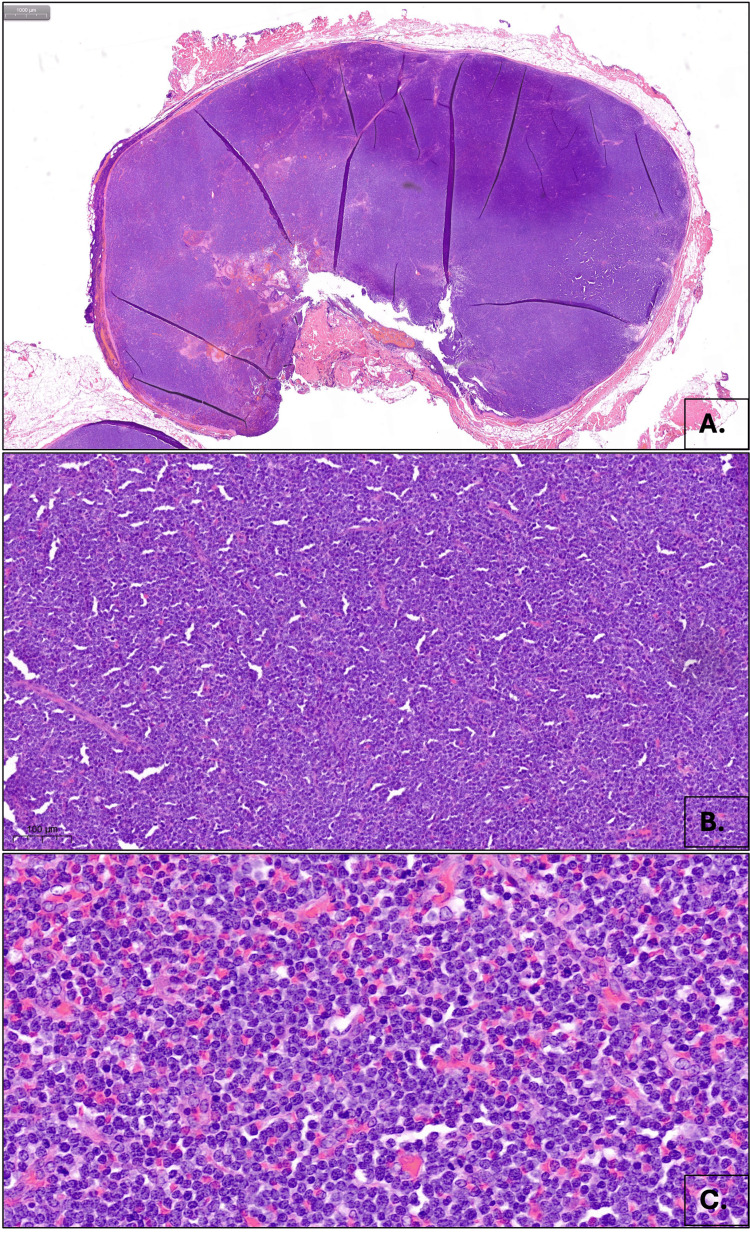
Extramedullary involvement in MPAL. A. Diffuse effacement of lymph node architecture, at 20x magnification, B. Sheets of medium to large monomorphic cells, at 200x magnification. C. Cytologic features demonstrating dispersed chromatin, inconspicuous nucleoli, and high mitotic index, at 600x magnification. MPAL: mixed phenotype acute leukemia.

Histopathologic examination demonstrated complete effacement of the normal lymph node architecture by a diffuse proliferation of medium to large monomorphic cells arranged in sheets. The neoplastic cells exhibited dispersed chromatin, inconspicuous nucleoli, and a high mitotic index (six mitoses per high-power field). Scattered residual secondary germinal centers were noted at the cortex (Figure 1A-C).

**Figure 2:**
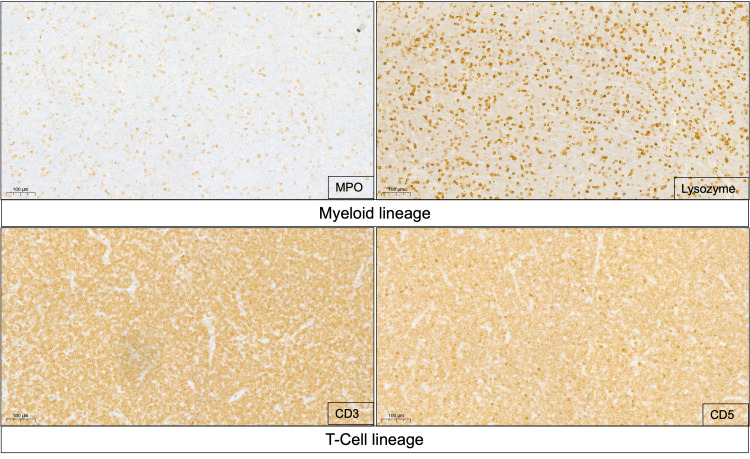
MPAL Myeloid/T-cell neoplastic lymph node infiltrate. MPO and lysozyme-positive staining markers of myeloid lineage. CD3 and CD5 positive staining markers of T cell lineage. MPAL: mixed phenotype acute leukemia.

Immunohistochemistry of the excised lymph node revealed that the neoplastic cells expressed CD34, c-KIT (CD117), CD3, CD4, CD5, CD7, myeloperoxidase (MPO, partial), and lysozyme. TdT was only expressed on rare cells, supporting a diagnosis of extramedullary involvement by T/Myeloid MPAL (Figure 2 and Table 1).

**Table 1:** Immunohistochemical Profile of Occipital Lymph Node Excisional Biopsy Summary of immunohistochemical findings highlighting the aberrant co-expression of T-lineage and Myeloid markers in the neoplastic blast population. The expression of cytoplasmic CD3 and MPO, along with immature markers (CD34, CD117), supports the MPAL (T/Myeloid type) diagnosis in accordance with WHO 5th edition criteria. Background markers are included for reference and confirmation of preserved non-neoplastic elements. MPAL: mixed phenotype acute leukemia; MPO: myeloperoxidase; CMYC: c-MYC (a proto-oncogene protein; assessed for overexpression via immunohistochemistry); BCL: BCL-2 and BCL-6 (B-cell lymphoma 2 and B-cell lymphoma 6 proteins, respectively; both assessed via immunohistochemistry); MUM1: multiple myeloma oncogene-1; PAX5: paired box 5; EBER: Epstein-Barr virus-encoded RNA (detected via fluorescence in situ hybridization (FISH)).

Marker	Result
CD3	Positive (cytoplasmic)
CD4	Positive
CD5	Positive
CD7	Positive
CD34	Positive
CD117 (CKIT)	Positive
MPO	Positive (subset)
Lysozyme	Positive (subset)
CMYC	Overexpressed
KI-67	~100%
BCL2	Positive (diffuse)
CD10	Negative
CD15	Positive (rare)
CD20	Positive (residual B cells)
CD21	Positive (residual FDC meshwork)
CD30	Negative
CD68	Positive
CD138	Positive (rare)
Cyclin D1	Negative
MUM1	Positive
PAX5	Positive (residual)
CD8	Positive (rare)
EBER (EBV ISH)	Negative

**Figure 3:**
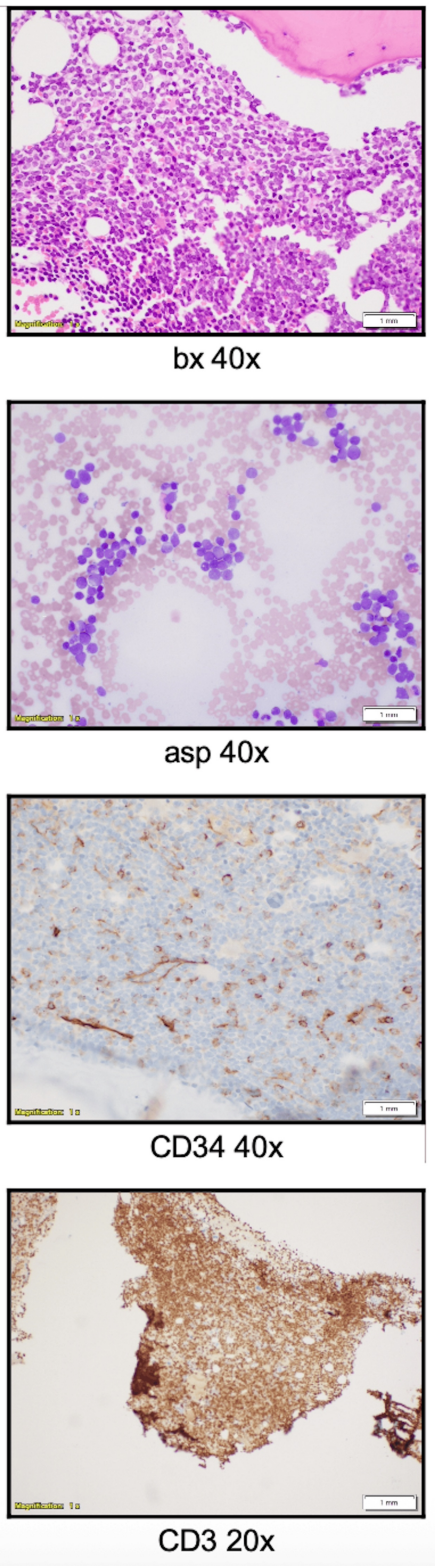
Bone marrow biopsy showing blast population expressing CD34 and cytoplasmic CD3.

A bone marrow biopsy demonstrated hypercellularity (30%) with 77% blasts, an immature infiltrate, and residual trilineage hematopoiesis. Immunostains confirmed extensive marrow infiltration by CD3+, CD5+, and CD2+ cells (90% of all cells). Additionally, approximately half of the abnormal cells (20-30%) expressed CD34, CD117, and TdT, further supporting a T/Myeloid MPAL phenotype (Figure 3).

Flow cytometry of the bone marrow aspirate revealed a blast population (22.4%) expressing CD34, cytoplasmic CD3, partial CD117, partial MPO, CD7, and CD56, while being negative for TdT. 

Cytogenetic and molecular studies identified a BCR-ABL1 [t(9;22)] gene rearrangement in 10% of cells by fluorescence in situ hybridization (FISH) analysis, with negative results for promyelocytic leukemia/retinoic acid receptor alpha (PML-RARA), core binding factor subunit beta (CBFB), lysine methyltransferase 2A (KMT2A) (MLL), and RUNX1T1/RUNX1. Cytogenetic analysis was inconclusive due to insufficient metaphases, and next-generation sequencing (NGS) (Tempus, Chicago, IL) did not identify significant pathogenic mutations in addition to the actionable mutation in BCR-ABL1.

Treatment

MPAL is particularly difficult to treat, in part because it is unclear whether it should be approached as acute myeloid leukemia (AML), acute lymphoblastic leukemia (ALL), or a combination of both [5]. This uncertainty complicates optimal clinical care. Based on the lack of clear treatment regimens, we decided to treat our patient with a combined regimen using fludarabine, cytarabine, idarubicin, granulocyte colony-stimulating factor (GCF), and venetoclax (FLAG-IDA + VEN). The FLAG-IDA + VEN regimen is an active treatment for AML, capable of producing high remission rates and facilitating transition to allogeneic hematopoietic stem cell transplantation (alloHSCT) when appropriate for most patients. A recent publication by the MD Anderson group revealed an overall response rate above 90% with the FLAG-IDA + VEN regimen [6,7]. Since our patient also presented with BCR-ABL positivity, we decided to add targeted therapy with dasatinib to prepare for an allogeneic hematopoietic stem cell transplant.

## Discussion

MPAL is a rare and diagnostically challenging entity. The T/Myeloid subtype represents a small fraction of cases and is often associated with an aggressive clinical course [1,4]. Extramedullary involvement in MPAL is rare, with nodal disease being an unusual presentation. This case underscores the importance of considering MPAL in patients presenting with generalized lymphadenopathy and circulating blasts.

An important differential diagnosis in this case is early T-cell precursor acute lymphoblastic leukemia (ETP-ALL), which shares significant immunophenotypic overlap with MPAL of T/Myeloid lineage. Both entities may express cytoplasmic CD3 and aberrant myeloid or stem cell markers, such as CD34, CD117, CD13, and CD33. However, ETP-ALL is defined by the absence of CD1a and CD8, weak or absent expression of CD5, and the presence of two or more myeloid or stem cell markers (e.g., CD13, CD33, CD34, CD117, HLA-DR, CD11b), without fulfilling the full criteria for myeloid lineage assignment [2]. In contrast, the diagnosis of MPAL requires definitive evidence of both T-lineage (cytoplasmic CD3) and myeloid-lineage (cytoplasmic myeloperoxidase or clear monocytic differentiation), which was evident in our case. Additionally, the presence of the* BCR::ABL1* fusion supports the classification of this leukemia as MPAL (T/myeloid) with a defined genetic abnormality, in accordance with the World Health Organization (WHO), fifth edition, and the International Consensus Classification (ICC) [1,8].

MPAL with* BCR::ABL1* fusion is particularly significant due to its prognostic and therapeutic implications. The optimal treatment strategy remains unclear, as MPAL with *BCR::ABL1* fusion is associated with poor responses to standard regimens and a high relapse rate [5,6]. *BCR::ABL1* rearrangements are among the most common genetic abnormalities in MPAL, present in approximately 14%-25% of the cases [1,9]. Their presence has significant therapeutic implications, as studies have shown improved outcomes when tyrosine kinase inhibitors (TKIs), such as imatinib or dasatinib, are incorporated into treatment regimens [9]. The management of MPAL remains particularly challenging due to its rarity, biological heterogeneity, and the lack of consensus on whether to treat it with AML, ALL, or hybrid protocols. Reported complete remission rates vary widely, ranging from 30% to 85%, and overall prognosis tends to be poorer than for other acute leukemias [9,10]. 

Several retrospective studies suggest that ALL-like regimens may offer improved outcomes, especially when combined with TKIs in *BCR::ABL1*-positive cases [9,10]. More recently, personalized approaches integrating cytogenetic, molecular, and immunophenotypic data, with allogeneic hematopoietic stem cell transplantation (allo-HSCT) in first remission when feasible, are increasingly being adopted. In the case we report, this evidence-based rationale informed the therapeutic approach: the patient was treated with dasatinib, a* BCR::ABL1*-targeted TKI, as a preparatory strategy for allo-HSCT (as detailed in the Treatment section), reflecting current practice patterns for this high-risk leukemia subtype.

This case highlights the diagnostic role of immunophenotyping and molecular studies in distinguishing MPAL from other acute leukemias and the importance of an integrated diagnosis between the medullary and the extramedullary findings. The presence of CD34, cytoplasmic CD3, and partial myeloid markers (CD117, MPO) was crucial in classifying this leukemia as T/Myeloid MPAL. Furthermore, the presence of TdT in rare cells, a marker typically seen in lymphoblastic leukemias, reinforces the unique immunophenotypic profile of this case.

Early recognition of extramedullary MPAL, particularly in the setting of lymphadenopathy, is essential for prompt diagnosis and treatment initiation. Further studies are needed to determine the most effective therapeutic strategies for MPAL with *BCR::ABL1* fusion, especially in cases with nodal involvement.

## Conclusions

This case emphasizes the importance of a multimodal approach to diagnosing MPAL, particularly in patients with atypical presentations such as generalized lymphadenopathy. The combination of histopathology, immunophenotyping, and molecular studies was essential in establishing the diagnosis. Given the aggressive nature of MPAL with *BCR::ABL1* fusion, early diagnosis and targeted therapy with TKIs are critical. Looking ahead, the integration of novel tools such as digital pathology platforms and machine learning algorithms may enhance the early detection of genomic abnormalities and assist in predicting treatment responses, potentially reducing turnaround times and accelerating the initiation of tailored therapies.
